# Deletion of the clock gene *Period2* (*Per2*) in glial cells alters mood-related behavior in mice

**DOI:** 10.1038/s41598-021-91770-7

**Published:** 2021-06-10

**Authors:** Tomaz Martini, Jürgen A. Ripperger, Jimmy Stalin, Andrej Kores, Michael Stumpe, Urs Albrecht

**Affiliations:** 1 Department of Biology, Faculty of Science and Medicine, University of Fribourg, 1700 Fribourg, Switzerland; 2 Department of Oncology, Microbiology and Immunology, Faculty of Science and Medicine, University of Fribourg, 1700 Fribourg, Switzerland

**Keywords:** Neuroscience, Glial biology, Astrocyte

## Abstract

The circadian clock regulates many biochemical and physiological pathways, and lack of clock genes, such as *Period* (*Per*) 2, affects not only circadian activity rhythms, but can also modulate feeding and mood-related behaviors. However, it is not known how cell-type specific expression of *Per2* contributes to these behaviors. In this study, we find that *Per2* in glial cells is important for balancing mood-related behaviors, without affecting circadian activity parameters. Genetic and adeno-associated virus-mediated deletion of *Per2* in glial cells of mice leads to reduced despair and anxiety. This is paralleled by an increase of the *GABA transporter 2* (*Gat2/Slc6a13*) and *Dopamine receptor D3* (*Drd3*) mRNA, and a reduction of glutamate levels in the nucleus accumbens (NAc). Interestingly, neuronal *Per2* knock-out also reduces despair, but does not influence anxiety. The change in mood-related behavior is not a result of a defective molecular clock, as glial *Bmal1* deletion has no effect on neither despair nor anxiety. Exclusive deletion of *Per2* in glia of the NAc reduced despair, but had no influence on anxiety. Our data provide strong evidence for an important role of glial *Per2* in regulating mood-related behavior.

## Introduction

Most organisms from cyanobacteria to humans have time-keeping mechanisms, termed circadian clocks, which allow adaptation to the 24-h day^42^. At the heart of this regulation lies a transcriptional-translational feedback loop, called the molecular clock. This clock is made up of a set of clock genes. In mammals, the positive arm of the clock mechanism is driven by the protein heterodimer of BMAL and CLOCK. This complex activates transcription of negative elements whose proteins Period (PER) and Cryptochrome (CRY) inhibit their own transcription by inactivating the BMAL/CLOCK transcriptional complex, thereby establishing an autoregulatory feedback loop^48^. The individual cellular clocks are orchestrated in an intricate manner to establish coherent systemic rhythms^18^ that are able to provide output signals to regulate various aspects of physiology and behavior. Some of these outputs regulate mood-related behavior through modulation of neurotransmitter synthesis, uptake and degradation^15,23,47^, and regulation of glucocorticoid signaling^37^. Absence or mutation of various clock genes have been associated with mood-related behaviors in mice and humans^15,23,34,38,50^. In particular, a whole-body mutation of the clock gene *Per2* revealed a manic phenotype in the Porsolt's forced swim test (FST), which was associated with reduced dopamine degradation, leading to increased dopamine levels in the nucleus accumbens (NAc)^23^. This is consistent with the view that the pharmacological manipulation of the monoaminergic system regulates mood, explaining in part the pathophysiology of depression^36^. However, current treatments for depression are often inefficient and require weeks of medication before the benefits of treatment can be observed^16^. Since major depression is a leading cause of disability in the western world and one of the main causes of death in adolescents, with a total of 800,000 suicides due to depression annually^22^, new approaches of tackling this debilitating condition are needed. An emerging new target for mood interventions are astrocytes^52^, which are in close metabolic and signaling interplay with neurons^2,32^. In order to ensure this interplay, the two cell populations need to be precisely synchronized to each other. Systemic and cellular synchronization is one of the main tasks of the circadian clock^18^ and therefore it is not surprising that astrocytes regulate rhythmic behaviors involving clock genes^6,8,26,49^. However, whether astrocytes that lack clock genes affect mood-related behaviors is not known. Since whole-body *Per2* mutant mice display changes in the reward system^1,47^ and despair perception^23^, we tested mice lacking the *Per2* gene in glial cells, including astrocytes.

To this end, we generated mice lacking *Per2* in glial fibrillary acidic protein (GFAP)-positive cells by cross-breeding *Per2* floxed mice with a *Cre* mouse line. In a second approach, we deleted *Per2* in GFAP positive cells of adult animals by delivering *Cre* with an adeno-associated virus to exclude developmental contributions to our experiments. Both models lacking *Per2* in *Gfap*-expressing cells (termed G*Per2* and vG*Per2*) were assessed for despair- and anxiety-related behavior using the FST and O-maze test, respectively. We found that mice from both models display a manic-like phenotype and are less anxious compared to control animals. In contrast to the whole-body *Per2* mutation, which displays a manic phenotype with reduced *monoamine oxidase A* (*Maoa*) and elevated dopamine levels^23^, *Maoa* was normal in the G*Per2* knock-out animals. However, we observed changes in the glutamatergic and GABAergic systems, as well as upregulation of the dopamine receptor D3.

## Results

### Deletion of *Per2* in glial cells by cross-breeding of mice

In order to study the importance of the *Per2* gene in glial cells, we crossed our floxed *Per2* (*Per2*^*fl/fl*^) animals^13^ with mice expressing the *Cre*-recombinase under transcriptional control of the human *Gfap* promoter (*GCre*)^53^. In families where the *Cre* was inherited from the maternal side, 58% of *Cre*-negative offspring (out of 80 progeny) showed germline recombination leading to total body *Per2* heterozygous deletion. In comparison, 0% of paternally inherited *Cre* (out of 260 progeny; Supplemental table 1) showed any germline recombination, as previously described^30^. Therefore, we used only male *Cre*-positive mice for matings with female *Per2*^*fl/fl*^ animals in order to obtain glial-specific deletion of *Per2* (*GCre*^+^ *Per2*^*fl/fl*^ termed G*Per2*).

**Figure 1:**
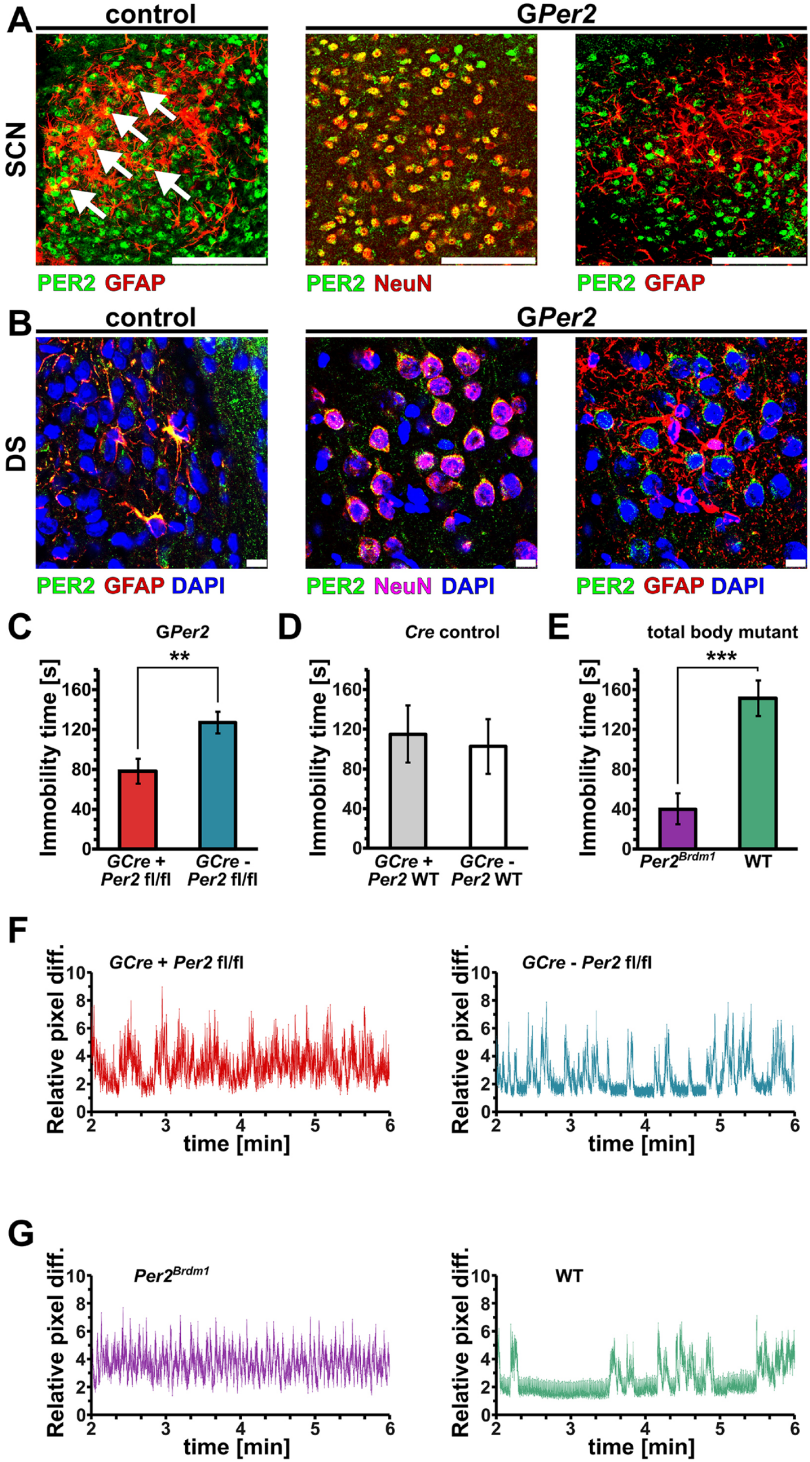
Genetic deletion of *Per2* in glia leads to depression resistant behavior. (**A**) Immunohistochemistry of sections from suprachiasmatic nuclei (SCN) of control (left panel) and G*Per2* mice (middle and right panels) collected at ZT12. Sections were incubated with antibodies against PER2 (green) and glial fibrillary acidic protein (GFAP), a glial marker (red, left and right panels), or NeuN (red, middle panel), a neuronal marker. White arrows in the control panel (left) indicate overlapping PER2 and GFAP signal (yellow) in glial cells, green indicates neuronal PER2 signal and red astrocytic GFAP. In G*Per2* sections, PER2 is mostly detected in neurons that are NeuN-positive, giving rise to the yellow color. In contrast, PER2 (green) is not seen in glial cells (red) of G*Per2* mice, indicating a glial specific deletion of PER2. Scale bar: 100 µm. (**B**) Immunohistochemistry of sections from the dorsal striatum (DS) of control (left panel) and G*Per2* mice (middle and right panels) collected at ZT12. Sections were stained with DAPI (blue) to reveal cell nuclei and were incubated with antibodies against PER2 (green) and GFAP (red, left and right panels) or NeuN (magenta, middle panel). The yellow color indicates overlapping signal of PER2 and GFAP in control animals (left panel) or PER2 and NeuN in neurons (middle panel). No yellow color in G*Per2* sections stained for PER2 and GFAP is observed. Scale bar: 10 µm. (**C**) Immobility time in the forced swim test (FST) of G*Per2* (*GCre*^+^ *Per2*^*fl/fl*^*,* red) and control (*GCre*^-^ *Per2*^*fl/fl*^*,* blue) animals are shown (n = 13 or 11, respectively, two-tailed t-test, **P < 0.01). (**D**) FST of the *Cre*-driver line (*GCre*^+^ *Per2 WT*) and the *Per2* control line (*GCre*^-^ *Per2 WT*) are shown (n = 6, two-tailed t-test, no significant difference is observed). (**E**) FST of *Per2* mutant (*Per2*^*Brdm1*^, purple) mice and their littermate controls (WT, green) show a significant difference as previously observed^23^ (n = 13 and 8, respectively, two-tailed t-test, ***P < 0.001). (**F**) Representative swimograms of G*Per2* (*GCre*^+^ *Per2 *^*fl/fl*^*,* red) and control (*GCre*^-^ *Per2* ^*fl/fl*^*,* blue) animals. Note the longer stretches of immobility in the control animals (right panel, blue). (**G**) Representative swimograms of *Per2* mutant (*Per2*^*Brdm1*^, purple) mice and their littermate controls (WT, green). Note the longer stretches of immobility in the control animals (right panel, green).

Next, we verified glial deletion of PER2 in brain tissue collected at zeitgeber time (ZT) 12 (where ZT0 is lights on and ZT12 is lights off) of G*Per2* mice using immunohistochemistry. Sections of the dorsal part of the suprachiasmatic nuclei (SCN) containing mainly arginine-vasopressin (AVP) neurons displayed immunoreactivity with a PER2 antibody (green) (Fig. 1A). In control animals co-staining with a GFAP antibody (red) showed partially overlapping signal with PER2 leading to yellow and orange staining, indicating PER2 expression in glial cells (white arrows, Fig. 1A, left panel). In G*Per2* mice, the red signal obtained with antibodies against the neuronal marker NeuN almost entirely overlapped with the green PER2 signal, resulting in the yellow/orange color (Fig. 1A, middle panel). This indicated that in the G*Per2* animals PER2 is still present in neurons. The PER2 (green) and GFAP (red) signal did not overlap in the G*Per2* animals and no yellow color was observed (Fig. 1A, right panel), strongly suggesting that PER2 was absent in glial cells. Hence, the green signal is due to neuronal PER2 expression, suggesting specificity of our approach.

To corroborate these observations, we performed immunohistochemistry in the striatum at ZT12 (Fig. 1B). The signals for PER2 (green) and GFAP (red) overlapped in the cytoplasm (yellow) and not in the nucleus (blue) of control animals (Fig. 1B, left panel). In G*Per2* animals, NeuN signal (magenta) overlapped with the PER2 signal (green) resulting in the yellow color (Fig. 1B, middle). Note that there was no green signal observed that could stem from glial PER2 expression. In the G*Per2* mice the PER2 signal (green) did not overlap with the GFAP signal (red) and PER2 appeared to be mainly present around the nuclei (blue) of neurons. Overall, our immunohistochemistry data indicate that PER2 was absent from glial cells of G*Per2* mice.

### G*Per2* mice display reduced despair

The glial *Per2* knock-out animals (G*Per2*) were tested for despair-based behavior, which is one of the important manifestations of depression. To this end, we used the forced swim test (FST) to assess time of immobility at ZT6^23^. We observed that G*Per2* mice (*GCre*^+^ *Per2*^*fl/fl*^) were significantly less immobile compared to control animals (*GCre*^*-*^* Per2*^*fl/fl*^) (Fig. 1C). The *Cre*-driver animals used for crossing (*GCre*^+^ *Per2*^*WT*^ and *GCre*^*-*^* Per2*^*WT*^) showed an immobility time comparable to the control animals (*GCre*^*-*^* Per2*^*fl/fl*^) (Fig. 1D). This illustrated that the lower immobility observed in the G*Per2* mice was specific to the lack of *Per2* in glial cells. As a positive control, we reproduced the lower immobility phenotype of *Per2*^*Brdm1*^ mutant mice (Fig. 1E) as described previously in male mice^23^, but this time using female animals to demonstrate that the phenotype was not gender specific. Figure 1F shows examples of primary data depicted as mobility over time (swimograms) with the corresponding color-coding. The data were obtained using a self-developed movement analysis software to determine mobility in an automated unbiased manner (see methods). It was evident that the control animals displayed long stretches of continuous baseline signal, which corresponded to long time stretches of immobility (Fig. 1F,G, right panels). These long stretches of immobility were very short or absent in both the G*Per2* (*GCre*^+^ *Per2*^*fl/fl*^) and *Per2*^*Brdm1*^ mutant mice (Fig. 1F,G, left panels). Taken together, our data show that lack of *Per2* in glial cells was sufficient to reproduce the manic-like phenotype observed previously in whole-body *Per2* mutant mice^23^. This suggests that *Per2* in glial cells may play an important role in the development of despair-based behavior contributing to depression.

### Deletion of *Per2* in glial cells using adeno-associated virus (AAV)-delivered *Cre* recombinase

The *Gfap*-Cre driver mouse line used above has been reported to potentially express *Cre* in some neuronal progenitor cells^24,40^. Furthermore, the *Cre* recombinase is most often inserted randomly into the genome and its correct position and number of copies are unknown. Additionally, *Cre* leakage was observed in some cases leading to unwanted recombination events^29,46^. Due to these potential problems and to exclude developmental effects in our deletion approach above, we delivered into adult mice a construct expressing the codon-improved *Cre*-recombinase (*iCre*) using engineered adeno-associated viruses (AAVs). An engineered AAV for efficient non-invasive gene delivery that can cross the blood–brain barrier (BBB) was used^11^.

**Figure 2:**
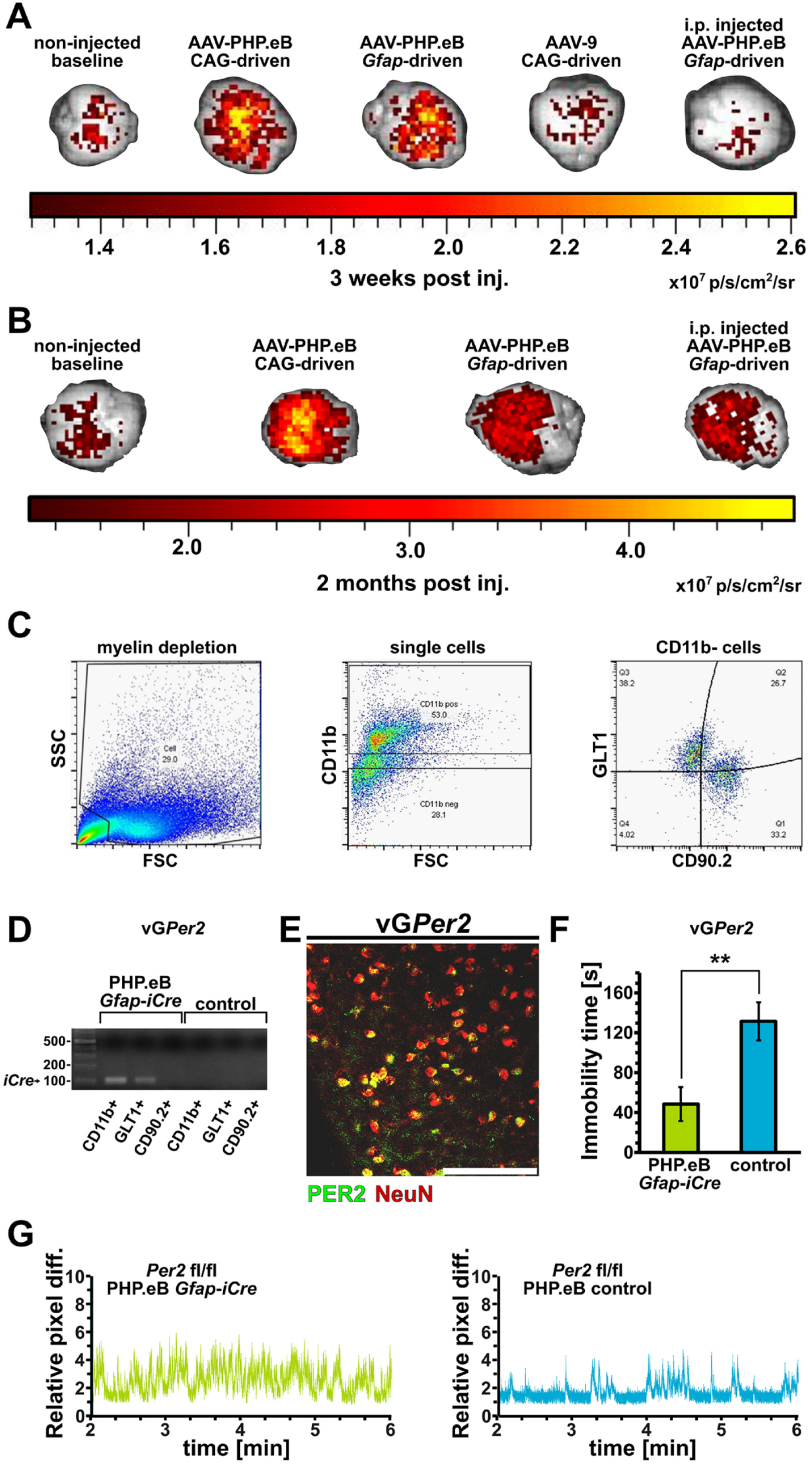
Adeno-associated virus (AAV)-mediated deletion of *Per2* in glial cells of adult mice leads to a depression resistant behavior. (**A**) Fluorescent imaging of whole brains 3 weeks after no injection (left), intravenous (i.v.) injection of the engineered AAV-PHP.eB, which can pass the blood–brain barrier (BBB), containing the general *CAG* driver (second from left) or the glial *Gfap* driver (middle). The second to last brain is from an animal with i.v. injected AAV9, which does not pass the BBB, containing the general *CAG* driver. The last brain (right) is from an animal injected intraperitoneally (i.p.) with the AAV-PHP.eB *Gfap*-driven construct. Note that only the brains of animals that received the AAV-PHP.eB i.v. display significant fluorescent signal after 3 weeks (orange and yellow color). (**B**) Fluorescent imaging of whole brains 2 months after injection of the AAV-PHP.eB. Note that the fluorescence is still maintained after 2 months post injection and that even the i.p. injected AAV-PHP.eB *Gfap* is showing signal in the brain now. (**C**) Sorting of neurons and astrocytes by flow cytometry from brain tissue including the nucleus accumbens (NAc). The left panel shows the removal of debris from a single cell suspension, showing the distribution of debris in the forward as well as in the side scatter (FSC and SSC, respectively). The middle panel shows the removal of CD11b^+^ cells (microglia) from the cell suspension. The CD11b^-^ cells (bottom half from middle panel) were then sorted into two distinct cell populations corresponding to astrocytes (GLT1^+^/CD90.2^-^) and neurons (CD90.2^+^/GLT1^-^) (right panel). (**D**) PCR analysis of astrocytes and neurons from the cell sorting. Microglia (CD11b^+^) as well as astrocytes (GLT1^+^/CD90.2^-^), but not neurons (CD90.2^+^/GLT1^-^) from PHP.eB *Gfap-iCre* infected animals express *iCre*, indicating that only glia and not neurons could express *iCre* in order to delete *Per2* in the *Per2*^*fl/fl*^ mice. All probes and markers were loaded on the same gel and the resulting photograph was not separated and recomposed. (**E**) Immunohistochemistry of vG*Per2* brain tissue from nucleus accumbens (NAc) isolated at ZT6. The signal for PER2 (green) mainly overlaps with neuronal NeuN signal (red) giving rise to the yellow color. Scale bar: 100 µm. (**F**) Immobility time in the forced swim test (FST) of vG*Per2* (PHP.eB *Gfap-iCre*, green) and control (PHP.eB control, blue) animals are shown (n = 7, two-tailed t-test, **P < 0.01). (**G**) Representative swimograms of vG*Per2* (AAV-PHP.eB *Gfap-iCre*, green) and control (AAV-PHP.eB control, blue) animals. Note the longer stretches of immobility in the control animals (right panel, blue).

This AAV-PHP.eB contained a vector expressing *iCre* under control of the human *Gfap* promoter with an enhanced green fluorescent protein (*eGfp*) as reporter. After intravenous (i.v.) injection via the lateral tail vein, fluorescence in the brain was detectable after 3 weeks in both the synthetic CAG-driven positive control^35^, as well as in the *Gfap*-driven constructs (Fig. 2A, red and yellow signals). No fluorescence and hence no BBB permeability was detected when using the natural variant AAV9 to deliver the CAG-driven control. Similarly, the non-injected control brain showed only baseline signal (Fig. 2A, brown). Intraperitoneal (i.p.) injection of the AAV-PHP.eB *Gfap*-driven *Cre*-construct also showed only baseline signal after 3 weeks. After 2 months, however, even i.p. delivery of the AAV-PHP.eB *Gfap*-driven construct resulted in detectable fluorescence, comparable to that of the i.v. injection (Fig. 2B, right), which is quite remarkable and is shown here to work for the first time. We termed the AAV mediated deletion of *Per2* in glial cells vG*Per2*.

Next, we wanted to check if the approach was specific for glial cells (astrocytes and microglia). We used flow cytometry and fluorescence-activated cell sorting to separate microglia (CD11b^+^), astrocytes (GLT1^+^) and neurons (CD90.2^+^). In a first step, the cells were depleted from debris (Fig. 2C, left panel), then the CD11b^+^ microglia were removed (Fig. 2C, middle panel) and subsequently the GLT1^+^ astrocytes were separated from the CD90.2^+^ neurons (Fig. 2C, right panel). The presence or absence of *iCre* was then determined by PCR on the different cell populations. We observed that the PHP.eB *Gfap*-*iCre* infected microglia (CD11b^+^) and astrocytes (GLT1^+^) expressed *iCre*. In contrast, neurons (CD90.2^+^) and non-infected controls did not express *iCre* (Fig. 2D, full size gel in Suppl. Figure 1A). Unfortunately, the sensitivity was not sufficient to reliably detect *Per2* expression in WT non-infected controls using this approach. However, immunohistochemistry on nucleus accumbens tissue isolated at ZT6 confirmed that PER2 protein could still be detected in neurons, while outside of neurons PER2 was barely or not detected (Fig. 2E).

### vG*Per2* mice display reduced despair

The animals with intravenously (i.v.) applied AAV-PHP.eB, which mediated the deletion of *Per2* in glial cells (vG*Per2*), were subjected to the FST in order to assess their immobility in this despair-based behavioral test. We observed that vG*Per2* (PHP.eB *Gfap-iCre*) mice showed significantly lower immobility times compared to control animals, which were injected with a comparable virus, but lacking *iCre* (PHP.eB control) (Fig. 2F). Examples of swimograms illustrating swimming behavior between minute 2 to 6 are shown (Fig. 2G, Suppl. Figure 2). Longer resting bouts were only detected in control animals (Fig. 2G, right panel, Suppl. Figure 2). This was consistent with the result we obtained by cross-breeding *Per2* floxed with *Gfap*-*Cre* mice (Fig. 1C, F, G), indicating that the phenotype can be reproduced by deleting *Per2* in glial cells of the adult animal. Hence, developmental processes are very unlikely to be responsible for this reduced despair phenotype.

### G*Per2* and vG*Per2* mice show reduced anxiety-like behavior in the O-maze

**Figure 3:**
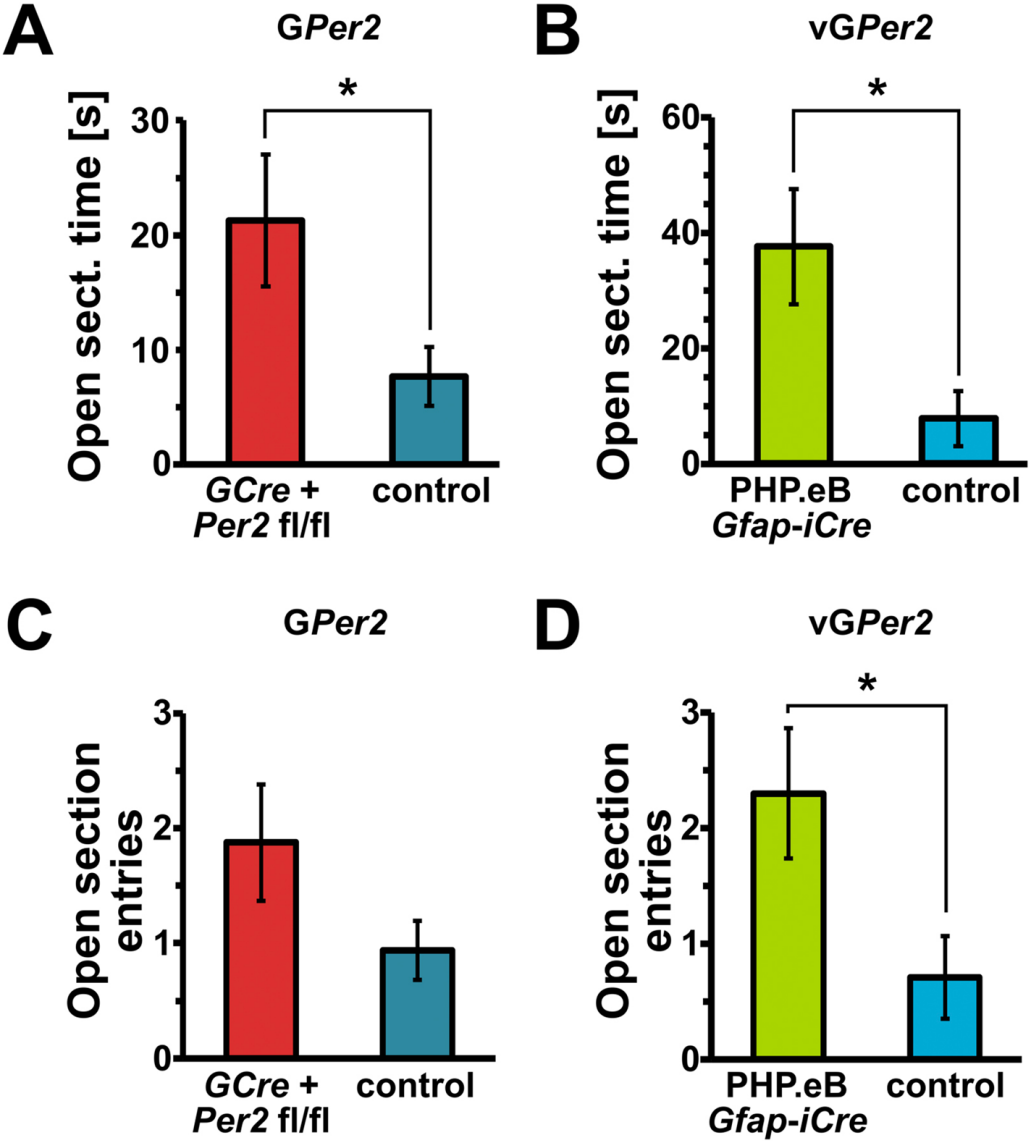
Reduced anxiety of G*Per2* and vG*Per2* animals in the elevated O-maze. (**A**) Time spent in the open area of the O-maze is longer in G*Per2* (*GCre*^+^ *Per2*^*fl/fl*^, red) animals compared to controls (blue) (n = 16 and 18, respectively, two-tailed t-test, *P < 0.05). (**B**) Time spent in the open area of the O-maze is significantly longer in vG*Per2* (AAV-PHP.eB *Gfap-iCre*, green) compared to controls (blue) (n = 6 and 7, respectively, two-tailed t-test, *P < 0.05). (**C**) Entries into the open section of the O-maze are not significantly different between the G*Per2* animals (red) and their controls (blue) (n = 16 and 18, respectively, two-tailed t-test, *P* = 0.15). (**D**) The number of entries into the open section of the O-maze is higher in vG*Per2* animals (green) compared to their controls (blue) (n = 6 and 7, respectively, two-tailed t-test, *P < 0.05).

Depression is a complex state and in addition to despair-related aspects also involves features of anxiety. Therefore, we tested the G*Per2* as well as the vG*Per2* animals in the elevated O-maze and measured how much time they spent in the open area of the maze and how many times they entered the open part. We observed that both the G*Per2* as well as the vG*Per2* animals spent more time in the open section of the O-maze compared to control animals (Fig. 3A, B). Interestingly, both G*Per2*, as well as vG*Per2* mice, displayed a tendency to explore the open sections more frequently (Fig. 3C, D). These observations indicate a reduced anxiety level in mice that lack *Per2* in glial cells.

### G*Per2* mice display normal circadian parameters

**Figure 4:**
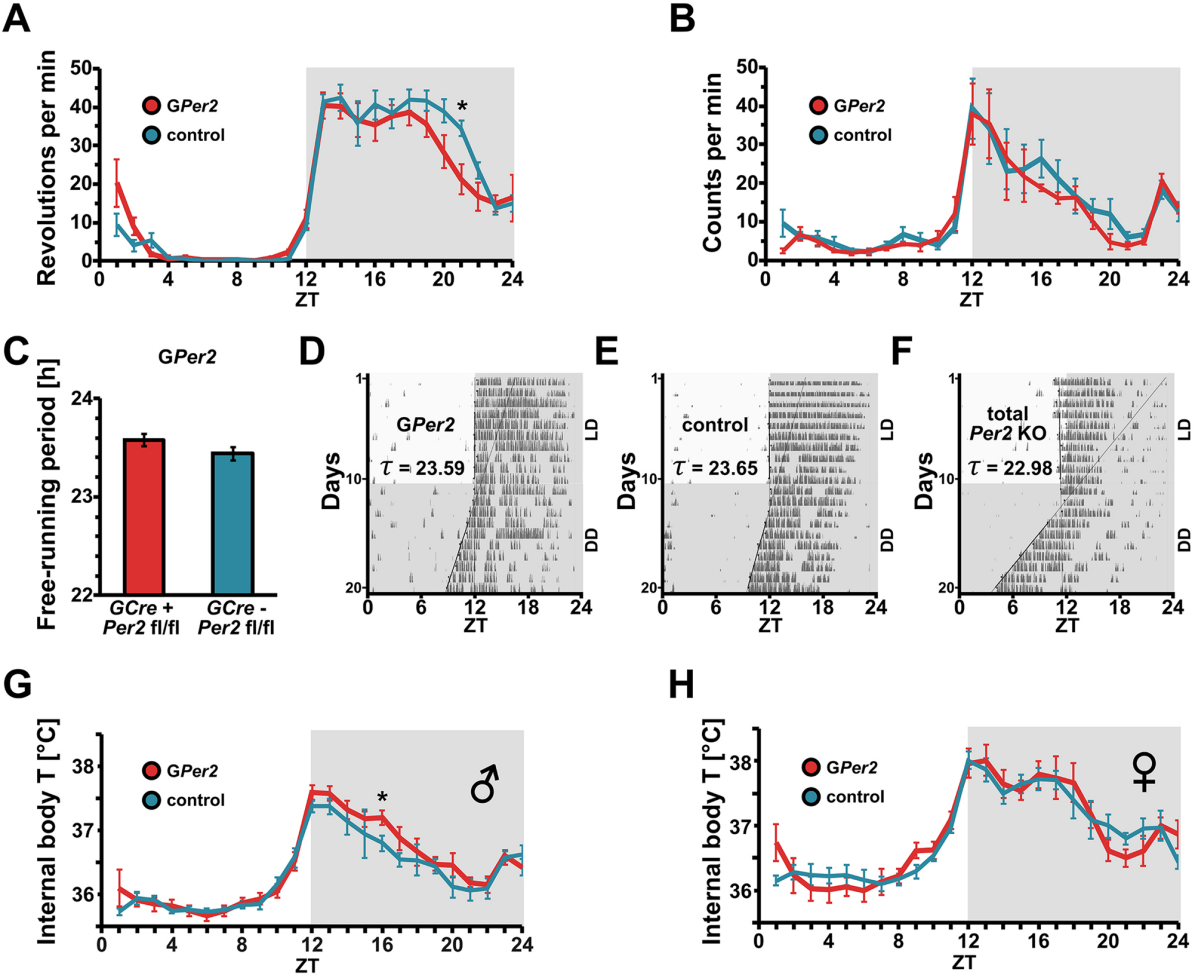
G*Per2* mice have a normal circadian clock. (**A**) Wheel-running activity profile of G*Per2* (*GCre*^+^ *Per2*^*fl/fl*^*,* red) and control (*GCre- Per2*^*fl/fl*^*,* blue) mice. The profiles are almost identical with a slight reduction of activity in G*Per2* animals at zeitgeber time (ZT) 21. (**B**) General activity pattern of G*Per2* (red) and control (blue) mice. The two profiles are not significantly different. (**C**) Circadian period of G*Per2* (red) and control (blue) mice. No significant difference was observed between the two genotypes (n = 6, two-tailed t-test, *P* > 0.05). (**D**) Representative wheel-running actogram of a G*Per2* animal. Black vertical marks represent activity in the wheel. Upper part shows the activity under an LD 12:12 cycle. Lower part shows the activity under constant darkness conditions (DD). (**E**) Representative wheel-running actogram of a control animal. (**F**) Representative wheel-running actogram of a whole-body total *Per2* knock-out animal. (**G**) Body temperature profile of male G*Per2* (red) and control (blue) mice under LD conditions (n = 6). (**H**) Body temperature profile of female G*Per2* (red) and control (blue) mice under LD conditions (n = 6).

Since G*Per2* mice display less immobility in the FST and swim more, we tested these animals for the circadian parameters of total activity, circadian period and body temperature fluctuation. We measured running-wheel revolutions in the cages of G*Per2* and control animals and observed identical activity profiles with low activity during the 12 h light phase and high activity during the 12 h dark phase (Fig. 4A). General activity was measured via an intraperitoneally implanted transmitter that was traced by a detector plate under the floor of the cage. Comparable to the wheel-running activity (Fig. 4A), no difference between the genotypes was observed (Fig. 4B). However, the activity in the second half of the dark phase (ZT16-22) was for both genotypes higher in the wheel-running assessment compared to the general activity pattern. These results show that the reduced immobility time of G*Per2* animals in the FST was not due to a higher activity level compared to controls and, hence, the higher activity in the FST is related to despair rather than to general activity.

Using the wheel-running activity data, we also determined the circadian period (τ) of the G*Per2* and total *Per2* knock-out (KO) animals under constant darkness conditions. The circadian period of G*Per2* mice was normal and not significantly different from control animals (Fig. 4C, D, E), indicating that loss of *Per2* in glial cells does not affect circadian period. In contrast, whole-body *Per2* KO mice displayed a period shorter than 23 h (Fig. 4F), consistent with previous findings^13,51^.

Next, we assessed body temperature over 24 h under a 12 h light : 12 h dark cycle (LD 12:12). No significant differences in the body temperature profiles were observed between G*Per2* and control animals, in both males (Fig. 4G) as well as females (Fig. 4H). This is consistent with the phenotype in *Per2*^*Brdm1*^ whole body mutant mice at 21 °C^12^ and liver, neuronal and total body *Per2* knock-out animals^13^.

Taken together, our data illustrate that loss of *Per2* in glial cells does not phenocopy all the characteristics of *Per2* whole-body KO animals, such as a shortening of the free-running period. This highlights a specific role of glial *Per2* in mood-related behaviors.

### Molecular changes in G*Per2* mice

Mood-related behaviors including depression are regulated by a number of different brain nuclei and regions^4,5,7,19,20,54^. We investigated three of those brain regions, the nucleus accumbens (NAc), the medial prefrontal cortex (mPFC) and the amygdala (AMY), as well as the hypothalamus (HYP) as a control brain region. The aforementioned brain regions also showed altered activity in response to swim stress^19^. We focused our attention on expression of genes involved in the synthesis, reuptake and degradation of monoamine neurotransmitters, as well as on genes involved in the clearance of glutamate and GABA from the synaptic cleft, because some of these processes were observed to be altered in *Per2* mutant mice^23,47^. Special attention was given to astrocyte-specific genes (Supplemental table 2).

**Figure 5:**
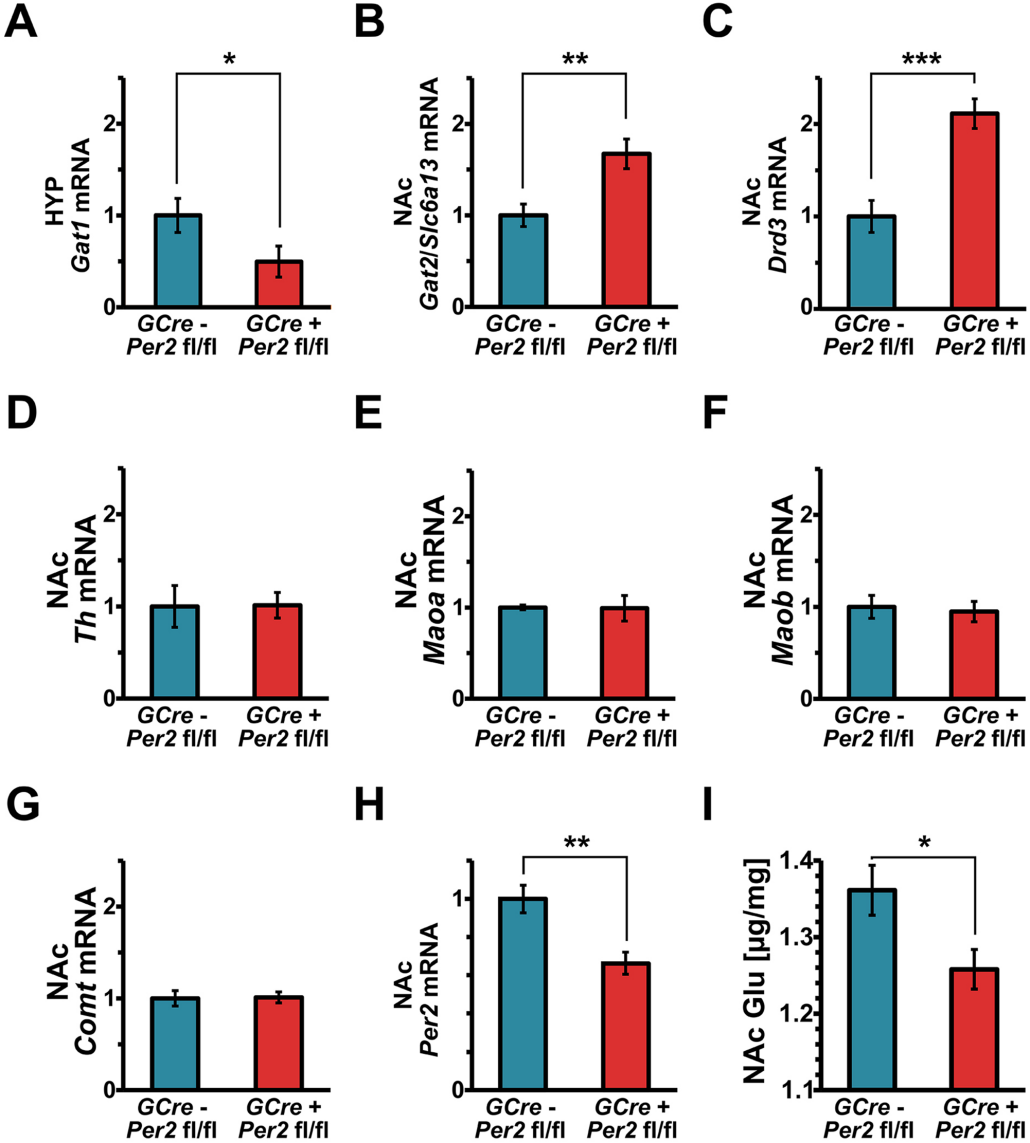
Molecular changes in G*Per2* mice. (**A**) Expression of *GABA transporter 1* (*Gat1*) mRNA in the hypothalamus (HYP) of G*Per2* (*GCre*^+^ *Per2*^*fl/fl*^*,* red) control (*GCre*^-^ *Per2*^*fl/fl*^*,* blue) at ZT2 (n = 3, two-tailed t-test, *P < 0.05). (**B**) Expression of *GABA transporter 2* (*Gat2/Slc6a13*) mRNA in the nucleus accumbens (NAc) of G*Per2* (red) and control (blue) animals (n = 6, two-tailed t-test, **P < 0.01). (**C**) Expression of *dopamine receptor D3* (*Drd3*) mRNA in the NAc of G*Per2* (red) and control (blue) animals (n = 6, two-tailed t-test, ***P < 0.001). (**D**) Expression of *tyrosine hydroxylase* (*Th*) mRNA in the NAc of G*Per2* (red) and control (blue) animals (n = 6, two-tailed t-test, *P* > 0.05). (**E**) Expression of *monoamine oxidase A* (*Maoa*) mRNA in the NAc of G*Per2* (red) and control (blue) animals (n = 6, two-tailed t-test, *P* > 0.05). (**F**) Expression of *Maob* mRNA in the NAc of G*Per2* (red) and control (blue) animals (n = 6, two-tailed t-test, *P* > 0.05). (**G**) Expression of *catechol-O-methyltrasferase* (*Comt*) mRNA in the NAc of G*Per2* (red) and control (blue) animals (n = 6, two-tailed t-test, *P* > 0.05). (**H**) Reduction of *Per2* in G*Per2* mice (red) compared to controls (blue). (**I**) Amount of glutamate (Glu) in the NAc of G*Per2* (red) and control (blue) animals at ZT6 (n = 5, two-tailed t-test, *P < 0.05).

In the hypothalamus (HYP), *GABA transporter 1* (*Gat1*/*Slc6a1*) mRNA was significantly decreased in G*Per2* (*GCre*^+^ *Per2*^*fl/fl*^) compared to control (*GCre*^*-*^* Per2*^*fl/fl*^) animals (Fig. 5A). In the nucleus accumbens (NAc), mRNA expression of *GABA transporter 2* (*Gat2*/*Slc6a13*) and *dopamine receptor D3* (*Drd3*) was increased in G*Per2* (*GCre*^+^ *Per2*^*fl/fl*^) compared to control (*GCre*^*-*^* Per2*^*fl/fl*^) mice (Fig. 5B, C). In contrast, *Gat1/Slc6a1* and *Gat3/Slc6a11* were unaltered (Supplemental table 1). Interestingly, genes coding for enzymes involved in monoamine synthesis, such as *tyrosine hydroxylase* (*Th*) and monoamine degradation, such as *monoamine oxidases A* (*Maoa*) and *B* (*Maob*), as well as *catechol-O-methyltrasferase* (*Comt*) were similar between the two genotypes in the NAc (Fig. 5D-G), while *Per2* mRNA was significantly reduced in G*Per2* mice (Fig. 5H). These results suggest that *Per2* in glial cells is involved in the regulation of GABA signaling, rather than the regulation of monoaminergic signaling. This was further underlined by our findings on the analysis of neurotransmitters. We found that glutamate (Glu) levels were significantly decreased in the NAc of G*Per2* (*GCre*^+^ *Per2*^*fl/fl*^) mice (Fig. 5I). This difference, however, was not significant in the other brain areas investigated (dorsal striatum (DS), medial prefrontal cortex (mPFC), Supplemental table 3). We did also not observe changes in GABA and glutamine in the NAc, DS and mPFC (Supplemental table 3).

Taken together, our molecular studies examining 4 regions of interest suggest that lack of *Per2* in glia affects mostly the NAc, and there the signaling pathways involving GABA and glutamate, as well as signaling via DRD3. NAc is therefore likely one of the regions involved in the observed behaviour.

### Deletion of *Per2* in glial cells of the NAc is sufficient to elicit a reduced despair phenotype

**Figure 6:**
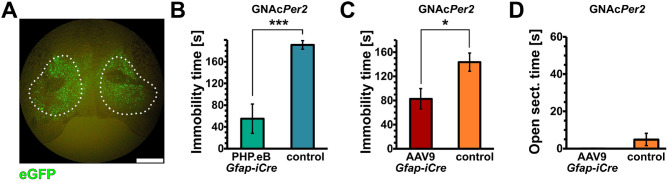
Deletion of *Per2* in glia of the NAc evokes reduced despair but has no effect on anxiety-related behavior. (**A**) Injection of the AAVs into the NAc as revealed by the eGFP reporter fluorescence. White dotted area delineates the NAc. Scale bar: 1 mm. (**B**) Immobility time in the forced swim test (FST) of PHP.eB *Gfap-iCre* (green) and non-injected control (*Per2*^*fl/fl*^*,* blue) animals is shown (n = 4 or 5, respectively, two-tailed t-test, ***P < 0.001). (**C**) FST of AAV9 *Gfap-iCre Per2*^*fl/fl*^ (red) and *Gfap*-*iCre Per2*^WT^ littermate control (orange) NAc injected mice is shown (n = 7 or 8, two-tailed t-test, *P < 0.05). Deletion of glial *Per2* in the NAc with both types of AAV capsid, but same expression cassette, appears to be sufficient to evoke reduced despair. (**D**) Time spent in the open area of the O-maze is similar in both NAc injected AAV9 *Gfap-iCre* and AAV9 control animals, suggesting no effect of *Per2* glial deletion in the NAc on anxiety-related behavior (n = 7 or 8, two-tailed t-test, *P* > 0.05).

Since we observed most of the molecular changes in the NAc of G*Per2* animals, we wondered whether deletion of *Per2* in glial cells of the NAc alone could elicit the phenotypes described above. Therefore, we injected our viral vectors directly into the NAc of *Per2*^*fl/fl*^ mice (Fig. 6A) using a stereotactic injection apparatus and termed the animals GNAc*Per2*. We saw that both AAV vectors, the PHP.eB *Gfap-iCre* as well as the AAV9 *Gfap-iCre* significantly reduced immobility time in the FST (Fig. 6B, C). The results were comparable to the observations in G*Per2* and the vG*Per2* animals, in which *Per2* was deleted in glial cells throughout the brain (Fig. 1 and 2). In contrast, we did not observe an effect on the time spent in the open section of the O-maze when AAV9 *Gfap-iCre* was injected into the NAc (Fig. 6D), suggesting that glial *Per2* in the NAc was not involved in the regulation of anxiety-related behavior.

Taken together, our data provide evidence that *Per2* expression in glial cells of the NAc is responsible for the regulation of despair-based behavior.

### The phenotype of G*Per2* mice is not the result of a defective glial molecular clock

**Figure 7:**
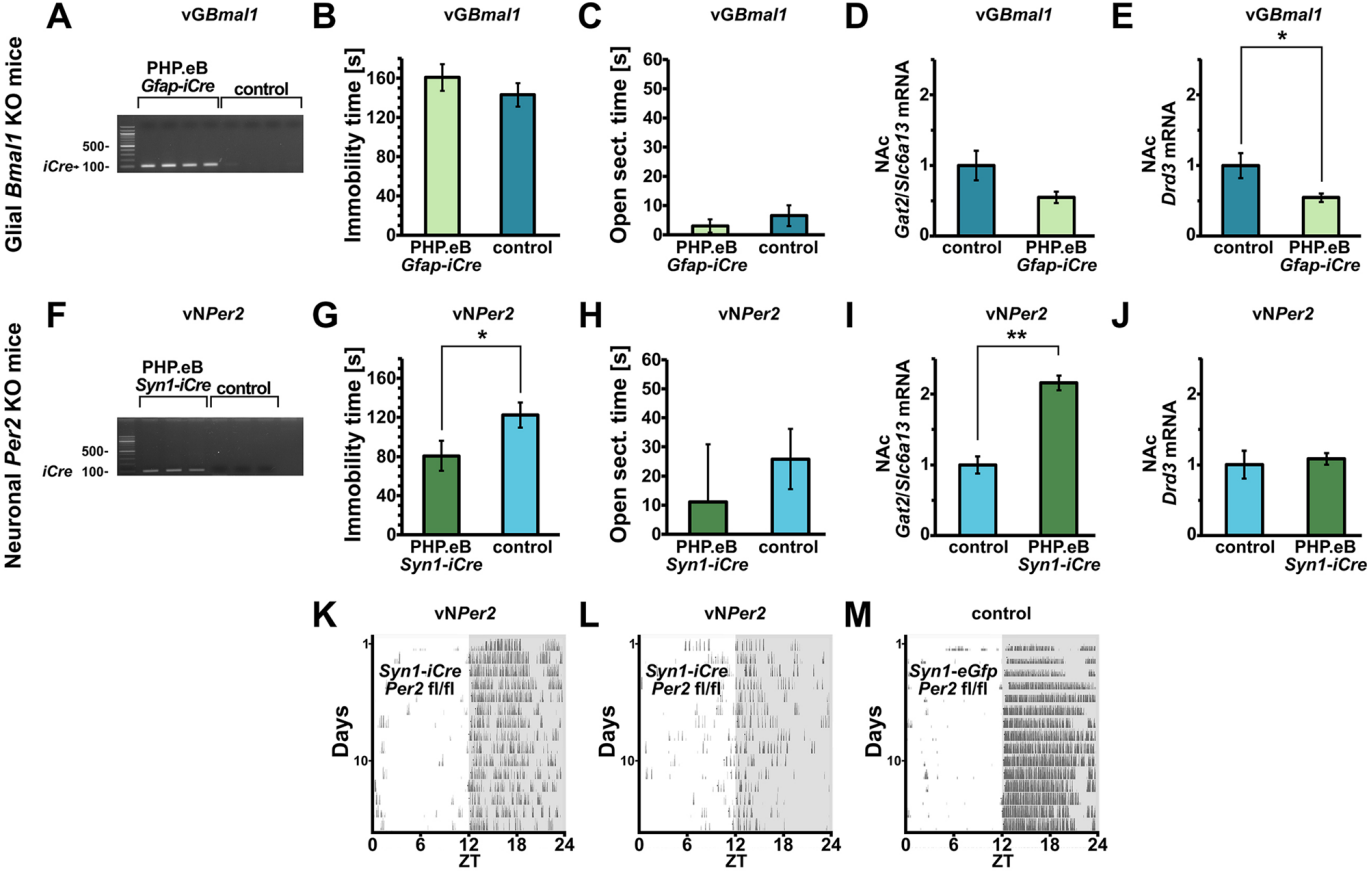
Deletion of *Bmal1* in glia does not influence despair- or anxiety-related behavior, while neuronal *Per2* deletion influences despair. (**A**) Agarose gel showing the expression of intravenously viral-delivered *iCre* in total NAc tissue of *Bmal1*^*fl/fl*^ and control animals (n = 4). All probes and markers were loaded on the same gel and the resulting photograph was not separated and recomposed. (**B**) Glial *Bmal1* KO mice do not show a difference in the FST (n = 8) or (**C**) O-maze (n = 8). Their NAc expression of (**D**) *Gat2* is not significantly reduced, whereas (**E**) *Drd3* expression is significantly lower in the KO group (n = 7 or 8, two-tailed t-test, *P < 0.05). (**F**) Agarose gel showing the presence of intravenously viral-delivered *iCre* in total NAc tissue of *Per2*^*fl/fl*^ and control animals (n = 3). All probes and markers were loaded on the same gel and the resulting photograph was not separated and recomposed. In comparison to vG*Bmal1*, vN*Per2* mice have (**G**) a reduced immobility time in the FST (n = 11 and 12, respectively, two-tailed t-test, *P < 0.05), but (**H**) show no difference to controls injected with an equivalent virus, but lacking *iCre*, in the O-maze (n = 11 and 12, respectively). (**I**) Their *Gat2*/*Slc6a13* expression is upregulated (n = 3, two-tailed t-test, **P < 0.01), while (**J**) the *Drd3* levels are unaltered in the NAc (n = 3). (**K**) A representative vN*Per2* mouse shows reduced locomotor activity in a wheel-running cage, as represented with an actogram, where each line represents a day of activity, the latter illustrated with the height and frequency of vertical lines. (**L**) Some KO mice also showed difficulties entraining to the light cycle. (**M**) The mice injected with the control virus, which lacked *iCre*, showed normal locomotor activity.

To answer the question whether the observed phenotype of G*Per2* mice comes from a defect of the molecular clock in glia, we used the aforementioned viral approach to generate mice lacking glial *Bmal1* (vG*Bmal1*), an essential molecular clock gene (Fig. 7A, full size gel Suppl. Figure 1B). These mice showed no changes of immobility time in the FST (Fig. 7B) and no difference in time spent in the open section of the O-maze (Fig. 7C). *Gat2/Slc6a13* was not significantly decreased, while *Drd3* expression was significantly lower in the NAc (Fig. 7D-E). The behaviour of vG*Bmal1* animals shows that the phenotype observed in G*Per2* mice is not a direct result of a defect of the molecular clock, but rather a change in the output of the clock involving the *Per2* gene. This is probably related to the function of the PER2 protein as a nuclear receptor co-regulator^44^.

To test whether the observed reduced despair and anxiety are specific to glial *Per2* function, we produced mice lacking *Per2* in neurons by utilizing the PHP.eB vector for genetic manipulation in adult animals. We systemically delivered a construct in which with the *Synapsin 1* (*Syn1*) promoter drove expression of *iCre* in neurons of *Per2*^*fl/fl*^ mice, and we named these mice vN*Per2* (Fig. 7F, full size gel Suppl. Figure 1C).

To our surprise, these mice also exhibited a lowered immobility time in the FST (Fig. 7G), albeit to a smaller degree than vG*Per2* mice (Fig. 2). However, we did not observe any change in the O-maze test (Fig. 7H). Interestingly, these mice also showed an increase of *Gat2/Slc6a13*, but normal expression of *Drd3* in the NAc (Fig. 7I-J). *Syn1*-driven neuronal *Per2* knock-out in adult animals was also associated with low activity levels under LD conditions (Fig. 7K). Interestingly, some mice showed difficulties in entrainment to the LD light cycle (Fig. 7L) compared to normal activity patterns of mice injected with an equivalent virus lacking *iCre* (Fig. 7M). This was an observation that we did not expect, as cross-bred neuronal *Per2* KO mice (*Nestin*-driven *Cre*) show normal activity patterns under LD conditions^13^.

## Discussion

Mood-related behaviors including depression are determined by several and sometimes synergistic factors illustrating the multifactorial nature of these neurological disturbances. In addition to the molecular complexity of these processes, cellular heterogeneity and topological organization complicate the understanding of the functioning of the brain even further. Previous studies have shown that at the molecular level genes involved in the regulation of the circadian clock can affect mood-related behaviors in mice^15,23,34^ and most likely also in humans^28,38^. Anatomical and genetic tracing studies have also revealed particular brain regions to be involved in various aspects of mood regulation.

Our previous studies have implicated a role of the clock gene *Per2* in mood-related behavior involving the mesolimbic dopaminergic system^23^. However, the contribution of glial cells in this process is poorly understood^52^. Therefore, we were interested to investigate the role of glial *Per2*. We used three approaches to investigate this question. First, we used genetic tools to delete *Per2* specifically in glial cells, including astrocytes (Fig. 1). Second, we deleted *Per2* in glial cells of the adult animal via systemic application of an engineered AAV that could pass the BBB (Fig. 2). This experiment was performed to exclude potential developmental effects that could contribute to behavioral alterations in the genetic approach. Third, we deleted *Per2* specifically in glial cells of the NAc by injection of AAVs expressing *Cre* recombinase. All three approaches, the genetic as well as the AAV approaches revealed that absence of *Per2* in glial cells, in particular the NAc, reduced the immobility time of mice in the FST, a test that assesses despair-based behavior, one of the aspects of depression. Glial deletion of *Per2* resulted in more active swimming in the FST, correlating with a manic behavior as we have previously observed in whole-body *Per2* mutant mice^23^. Interestingly, pharmacologic glial ablation in the prefrontal cortex has been reported to increase immobility in the FST and as a consequence made the animals more depressive^5^. This phenotype is the opposite from what we observed in our G*Per2* and vG*Per2* mice and indicates that in our animal models glial cells are still functional and not eliminated. Hence, our observations are specific to glial *Per2* function and are very unlikely related to processes leading to glial cell death.

The processes affected by lack of *Per2* in glial cells are most likely not related to the circadian clock mechanism, because G*Per2* animals display no abnormalities in clock parameters such as period or activity distribution and body temperature fluctuations (Fig. 4). If the disturbance of the clock mechanism in astrocytes would play a major role, a desynchronization between astrocytes and neurons would be expected, which would ultimately lead to arrhythmic activity of mice under constant darkness conditions. This is, however, not what we observed (Fig. 4). Furthermore, deletion of *Bmal1* in glial cells did not affect immobility time in the FST (Fig. 7), suggesting that the clock itself does not cause the phenotype we observed in glial *Per2* knock-out animals. Therefore, we hypothesize that the phenotypes we describe here are probably related to PER2 output functions, e.g. as nuclear receptor co-regulator^44^. PER2 may potentially act as a nuclear receptor co-regulator, which would correlate with the altered levels of *Gat2/Slc6a13* and *Drd3* mRNA that we observed in the NAc (Fig. 5B). GABA transporters as well as *Drd3* have been implicated in mood-related behavior. *Drd3* has been shown to be regulated by the transactivator RORα and repressor REV-ERBα^25^, both of which bind to the same promoter elements (ROREs) and of which REV-ERBα is modulated by PER2^44^. However, the regulation of *Gat2/Slc6a13* remains elusive.

Since depression is not only related to despair, we also tested our animals in the elevated O-maze. This test addresses anxiety-related behavior, which is an important component of depression. We observed that both G*Per2* as well as vG*Per2* mice were less anxious, because they spent more time in the open area of the O-maze (Fig. 3). Interestingly, however, the animals with *Per2* deleted in the glia of the NAc only (GNAc*Per2*) did not show this phenotype (Fig. 6D). From this we conclude that *Per2* in glia of the NAc may not be important for anxiety-related behavior and specifically affects despair. Hence, glial *Per2* in other brain regions, such as the amygdala^17^, the bed nucleus of the stria terminalis (BNST)^41^ or the subgenual anterior cingulate cortex (sgACC)^3^, part of the medial prefrontal cortex (mPFC), may be involved in the modulation of anxiety. Interestingly, vN*Per2* mice showed no difference in anxiety perception (Fig. 7H) and it seems that the reduced anxiety is mediated mainly through glial *Per2*. This suggests that distinct brain regions and also different underlying mechanisms lead to reduced despair and anxiety in G*Per2* and vG*Per2* mice. Whereas the vN*Per2* mice also showed increased *Gat2* levels, as observed in G*Per2* mice, they had no alterations in *Drd3* expression.

We did not test anhedonia, another aspect of depression, because whole-body *Per2* mutant mice did not show any changes in the sucrose preference test^47^. Therefore, we assumed that glial *Per2* plays a subordinate or no role in the anhedonia aspect of depression.

Our previous studies describing whole-body *Per2* mutant mice highlighted a functional involvement of *Per2* in the regulation of monoamine degradation^23^. In this study, we find that glial *Per2* appears to play a role in the regulation of glutamate, GABA and dopaminergic signaling, but not monoamine metabolism (Fig. 5), at least not at the observed time-point. There is ample evidence that both, monoaminergic and GABAergic signaling regulate depressive disorders^31^, and *Per2* might be involved in both. If this hypothesis was correct, then we would expect that deletion of *Per2* in neurons would still affect mood-related behaviors. Our results are consistent with this view, because vN*Per2* mice show reduced immobility in the FST (Fig. 7F).

Alterations of glial function are considered to modify glutamate reuptake^14^, which may be the reason why we observed reduced glutamate levels in the NAc of our G*Per2* knock-out animals (Fig. 5I). Interestingly, however, we did not observe changes in expression of glutamate transporters *Eaat1* and *Eaat2* (Supplemental table 2). This is in contrast with our previous study investigating whole-body *Per2* mutant mice, which show decreased *Eaat1* levels^47^. Hence, it appears that lack of *Per2* function in neurons and astrocytes leads to changes in *Eaat1* expression, illustrating the complex interplay between neurons and astrocytes in the regulation of glutamate signaling and mood-related behavior.

Overall, our results provide evidence for a specific role of glial *Per2* in mood-related behavior, accompanied by dysregulation of components of the glutamatergic, GABAergic and dopaminergic signaling pathways.

## Materials and methods

All experiments were approved by the Bundesamt für Umwelt BAFU and the State Veterinarian of the Canton of Fribourg.

### Animals and housing

All mice were housed with food and water ad libitum in transparent plastic cages (267 mm long × 207 mm wide × 140 mm high; Techniplast Makrolon type 2 1264C001) with a stainless-steel wire lid (Techniplast 1264C116), kept in light- and soundproof ventilated chambers. All mice were entrained to a LD 12:12 cycle, and the time of day was expressed as zeitgeber time (ZT; ZT0 lights on, ZT12 lights off). Two- to four-month-old males and females were used for the experiments unless otherwise stated. Males were used for the FST, while females were used for the O-maze and mixed genders to monitor activity. Housing as well as experimental procedures were performed in accordance with the guidelines of the Schweizer Tierschutzgesetz and the Declaration of Helsinki. The state veterinarian of the Canton of Fribourg approved the protocols. The study was carried out in compliance with the ARRIVE guidelines.

Conditional glial *Per2* knock-out animals were generated using *Gfap*-*Cre* mice (Jackson lab FVB-Tg(GFAP-cre)25Mes/J, stock no. 004600, created by the laboratory of A. Messing) that were cross-bred with our *Per2* floxed animals (^13^, European Mouse Mutant Archive (EMMA) strain ID EM: 10,599, B6,129P2-Per2^tm1Ual^/Biat). The resulting GPer2 line was back-crossed to the C57BL/6 strain. The *Per2*^*Brdm1*^ mutant mice^51^ and their wild-type littermate controls were on a mixed 129 and C57BL/6 background.

### Viruses used and their application

The following viruses, produced by the Viral Vector Facility of the Neuroscience Center Zurich, were used: v95-PHP.eB (ssAAV-PHP.eB/2-hGFAP-EGFP-WPRE-hGHp(A)) as the control virus and v232-PHP.eB (ssAAV-PHP.eB/2-hGFAP-EGFP_iCre-WPRE-hGHp(A)) as the virus for recombination, v344-9 (ssAAV-9/2-shortCAG-chI[1 × (shm/rNS)]-EGFP-WPRE-SV40p(A)) and v25-PHP.eB (ssAAV-PHP.eB/2-CAG-EGFP_Cre-WPRE-SV40p(A)) as controls for BBB permeability determination, while stereotactic injections were also performed with constructs v95 and v232 in the AAV9 capsid. All viruses expressed the green fluorescent protein as a marker.

For intravenous delivery of viruses, mice were sedated and chemically restrained with an intraperitoneal injection of 40 mg per kg body mass ketamine and 0.15 mg per kg medetomidine in saline and placed on a heating pad, while hydrogel was applied to their eyes. We injected 10^11^ viral genomes per mouse in a total volume of 200 μl PBS via the lateral tail vein. The sedation was reversed with atipamezole in 5 times the dose of medetomidine.

Whole-brain fluorescent imaging was performed using the IVIS Lumina II (Caliper LifeSciences) and the accompanying Living Image software (version 4.2.0.14335). Settings: excitation 465 nm, filter GFP, exposure time 1 s, light level low, binning low/small.

### Forced swim test (FST)

The Porsolt’s forced swim test has been described in detail elsewhere^23,39^. Briefly, the mice were placed into a cylinder filled with water, where they were left for 6 min. The first 2 min were discarded as adaptation period, while the following 4 min were scored. The test was performed for four consecutive days. The first day is regarded as adaptation and was discarded. The following three days were manually scored for immobility time – the amount of time a mouse passively floated during the 4 min test window. An average immobility time for each mouse was calculated and then these were pooled according to the assigned experimental group. In the case of animals injected with the PHP.eB virus, the forced swim test was performed 4 weeks post injections^10^. All forced swim tests were performed at ZT 6, 6 h after lights were switched on in a 12 h light, 12 h dark environment^23^. The water temperature was always adjusted to 26 + /− 1 °C.

The FSTs were recorded from the side view. The automated forced swim test analysis was performed with a custom written program that evaluated the relative pixel difference in a rectangular area spanning the width of the swim tank and having the height equal to one sixth of the width, measured from the water surface towards the bottom of the tank. Each pixel in the test area was evaluated for changes between frames in red, green and blue on the decimal scale, the differences were averaged and divided by the test area, resulting in a graph that showed relative pixel change per area over time.

### O-maze

The anxiety-based O-maze test was performed on an elevated 5.5 cm wide circular runway with an outer diameter of 46 cm, which was divided into 4 sections, with 2 opposing closed sections. The time spent in the open sections was evaluated based on video recording. Entry into the open section was considered when the mouse entered it with all 4 paws. To avoid habituation to the test environment, a single test of 5 min was performed. Two mice with values more than 2 standard deviations from the group mean were excluded from the study as outliers: one mouse was removed from the G*Per2* group (276 s or 92% of the time in the open section) and one mouse from the corresponding control group (40 s in the open section). One mouse was also excluded from the vG*Per2* group (274 s or 91% of the time in the open section).

### Tissue isolation, gene expression analysis and neurotransmitter quantification

For both gene expression analysis and neurotransmitter quantification, fresh brain tissue was dissected and immediately submerged into liquid nitrogen. For the list of primers, please see Supplemental table 4. For gene expression analysis, RNA was isolated using the Macherey–Nagel RNA Plus kit and reverse transcribed using the Invitrogen SuperScript II. qPCRs were performed using the RotorGene 6000, with the KAPA Probe or KAPA SYBR master mix reagent.

### Surgical procedures

The surgical procedures were performed as previously described^33^. Briefly, mice were anesthetized with 80 mg per kg ketamine and 0.30 mg per kg medetomidine, while the anesthesia was reversed by atipamezole in 5 times the dose of medetomidine. Before surgical procedures, the depth of anesthesia was checked by an absence of a reflex when pinching the skin between the toes of the mouse 5–10 min after application of the anesthetic. In case of a reflex, a quarter of the initial dose of the anesthetic was additionally administered. For measurements of internal body temperature and general activity, a wireless biochip (VitalView system) was implanted into the abdominal cavity (Starrlife Sciences, VitalView Data Acquisition System, Instruction Manual, Software Version 5.1). Stereotactic injections were performed with a pulled glass pipette, which allowed injections of 2 × 200 nl of the virus bilaterally into the nucleus accumbens (NAc) with stereotactic coordinates of 1.60 anterior–posterior, + /- 1.00 medio-lateral and 4.50 dorso-ventral. Surgical procedures were followed by administration of carprofen at 10 mg per kg.

### Immunohistochemistry

Brains were harvested after mice were cardiovascularly perfused with saline and 4% PFA, and the tissue was left in PFA over night, and then transferred to 30% sucrose in PBS for two days for cryoprotection. 40 µm sections were cut using a cryostat and kept at -20 °C in an antigen preservation solution (1% m/m polyvinyl pyrrolidone in a 1 : 1 mixture of PBS and ethylene glycol) until subsequent steps of washing, blocking with permeabilization, antigen retrieval and antibody staining (anti-PER2, Alpha Diagnostic, cat. PER21-A, 1 : 200 dilution; anti-GFAP, Abcam, cat. ab53554, 1 : 500 dilution; anti-NeuN, Merck Millipore, cat. mab377, 1 : 250 dilution). The procedures have been described by our laboratory in detail elsewhere^9^. Images were acquired using a Leica SP5 confocal microscope.

### Wheel-running experiments, general activity and internal body temperature measurements

Wheel-running experiments were performed using custom built cages, according to local legislation on animal experimentation, which had a stainless-steel wire running wheel with a diameter of 11.5 cm. On the axis, a system with a magnet closed a switch for each wheel revolution. The data was digitalized using an interface from Actimetrics and the activity was recorded and processed using the ClockLab software version 6.0.54.

This allowed acquisition of activity patterns in LD conditions, as well as monitoring of the subjective day length under constant conditions. For this, the mice were entrained to LD conditions and then released into total darkness for a minimum of 10 days. The first three days were discarded as assimilation, while the subsequent 7 days were evaluated for a shift of activity onset on each consecutive day in order to give a measure of the length of the subjective day or free-running period, as previously described^27,33^.

Telemetrics was performed with the VitalView system, which is used for wireless measurements of general mouse activity, as well as internal body temperature, allowing monitoring of behavioural and physiological changes in free-running animals. For the biochip implantation, please see ‘Surgical procedures’. All the procedures have been described in detail elsewhere^33^.

### Flow cytometry

At the desired time-point, brain tissue was harvested, dissected, stored in a test tube and submerged into cooled isopentane. The samples, still in the isopentane bath, were then stored at − 80 °C^43^. For flow cytometry, the samples were diced with razor blades and digested under heat and agitation with the Neural Tissue Dissociation Kit (T) (130–093-231) from Miltenyi Biotech according to the manufacturer’s protocol. Single-cell suspensions were obtained by straining through a 70 µm mesh filter, after which the strained cells were washed twice in FACS buffer (PBS with 5% fetal calf serum and 5 mM EDTA). Then, myelin debris were removed using the Myelin Removal Beads II kit (130-096-731) from Miltenyi Biotech. Myelin-depleted cell suspensions were incubated with an anti-CD16/CD32 Fc-blocking antibody (BD Biosciences) for 30 min at 4 °C to avoid Fc receptor binding and then washed once with FACS buffer. The cells were stained with fluorophore-conjugated antibodies and analyzed on a MACSQuant flow cytometer (Miltenyi Biotech) or sorted using the FACSAria Fusion cell sorter (BD Biosciences). Flow cytometry analysis was based on viable and single-cell gaiting strategies, as previously described^45^. Antibodies targeted CD11b (fluorophore PECy7, ref. 552,850, BD Pharmingen), CD90.2 (fluorophore PE, ref., 130-102-489, Miltenyi Biotech) and GLT1 (fluorophore ATTO 633, ref., AGC-022-FR, Alomone lab), and were used in a 1:100 dilution.

## Supplementary Information

Supplementary Information.

## Abbreviations

G*Per2*: Constitutive glial *Per2* KOs (*Gfap*-*Cre*^+^ *Per2*^*fl/fl*^)
vG*Per2*: Viral-mediated glial *Per2* KO in adult animals (*Gfap*-*iCre* into *Per2*^*fl/fl*^ mice)
GNAc*Per2*: Nucleus accumbens glial *Per2* KO (*Gfap*-*iCre* into *Per2*^*fl/fl*^ mice)
vN*Per2*: Viral-mediated glial *Per2* KO in adult animals (*Syn1*-*iCre* into *Per2*^*fl/fl*^ mice)
vG*Bmal1*: Viral-mediated glial *Bmal1* KO in adult animals (*Gfap*-*iCre* into *Bmal1*^*fl/fl*^ mice
