# Supplementary material for: Deletion of the clock gene Period2 (Per2) in glial cells alters mood-related behavior in mice
Source: Sci Rep. 2021 Jun 10;11:12242. doi: 10.1038/s41598-021-91770-7 (PMC8192521; doi:10.1038/s41598-021-91770-7)
Supplement: Supplementary file 1 — Supplementary Information. [file 41598_2021_91770_MOESM1_ESM.pdf]

Deletion of the Clock Gene *Period2* (*Per2*) in Glial Cells Alters Mood-Related Behavior in Mice

Tomaz Martini [1], Jürgen A. Ripperger [1], Jimmy Stalin [2], Andrej Kores [1], Michael Stumpe [1], and Urs Albrecht [1, *]

[1] *Dept. of Biology, Faculty of Science and Medicine, University of Fribourg, 1700 Fribourg, Switzerland*

[2] *Dept. of Oncology, Microbiology and Immunology, Faculty of Science and Medicine, University of Fribourg, 1700 Fribourg, Switzerland*

* correspondence: urs.albrecht@unifr.ch

Supplemental Figure Legends

Supplemental Figure 1

Full size gels corresponding to A) Fig. 2D, B) Fig. 7A and C) Fig. 7F

Supplemental Figure 2

Representative swimograms of PHP.eB-mediated glial KO animals (*Per2*$^{fl/fl}$ *Gfap-iCre-eGfp*) on the final day of the forced swim test (green) vs. PHP.eB injected controls (*Gfap-eGfp*, blue) show a lack of longer stretches of immobility time in the KO group, with prolonged stretches of low activity in the control group.

Supplemental Figure 3

Bmal1 mRNA quantification in NAc tissue at ZT 2 shows that vG*Bmal1* mice (PHP.eB *Gfap-iCre*, green) have reduced *Bmal1* expression compared to control (blue) animals (n = 7 or 8, two-tailed t-test, * $p < 0.05$).

Supplemental Tables

Supplemental Table 1: Statistics of germline recombination in different families of G*Per2* mice.

A simple determination of the percent of mice with germline recombination was based on the frequency of the deleted *Per2* allele in *Cre*-negative offspring. This frequency was individually evaluated for families where the *Cre* carrier was the male or female breeding partner, respectively. Only families where both alleles were floxed in the mating partners were analyzed.

Supplemental Table 2: Gene expression analysis in the NAc, mPFC, AMY and HYP.

Changes in gene expression in different brain regions. The relative change was calculated by dividing the relative mRNA in the G*Per2* by that in the control group, and the group comparison was made with the two-tailed Student's t-test.

Supplemental Table 3: Glu, Gln and GABA quantification in the NAc, DS and mPFC.

Freshly isolated and flash-frozen tissue was used for total (intra- and extracellular) quantification of neurotransmitter content at ZT6. The results are given in both nanomoles and nanograms per mg of isolated tissue in control animals (C) and G*Per2* animals (KO). The group comparison was made with the two-tailed Student's t-test.

Supplemental Table 4: List of primers used for polymerase chain reaction (PCR)

The TM probes were synthesized with a 6-fluorescin (FAM)group at the 5’-end and a black hole quencher 1 (BHQ1) group at the 3’-end. All other primers were unmodified.

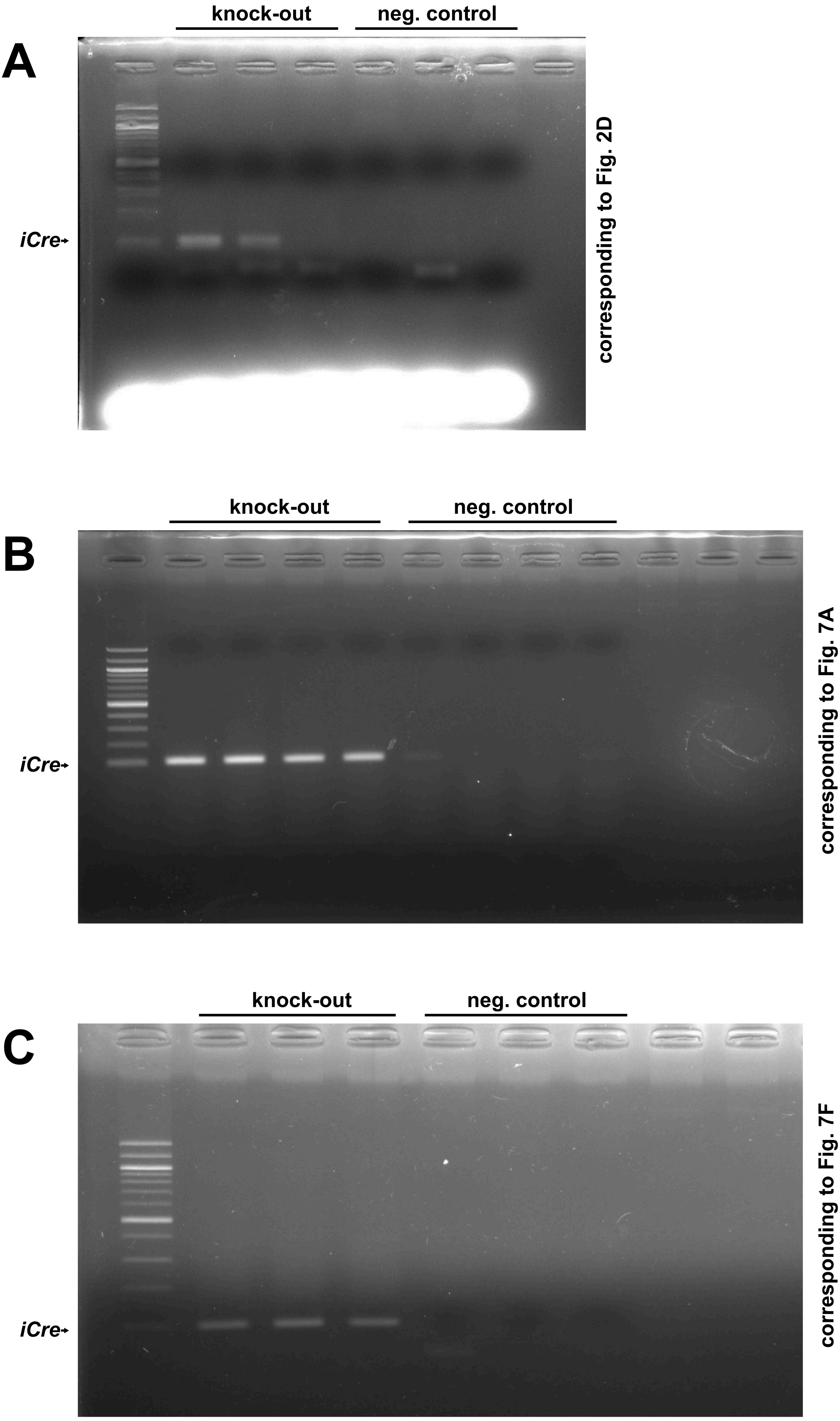
A
knock-out
neg. control
iCre
corresponding to Fig. 2D
B
knock-out
neg. control
iCre
corresponding to Fig. 7A
C
knock-out
neg. control
iCre
corresponding to Fig. 7F

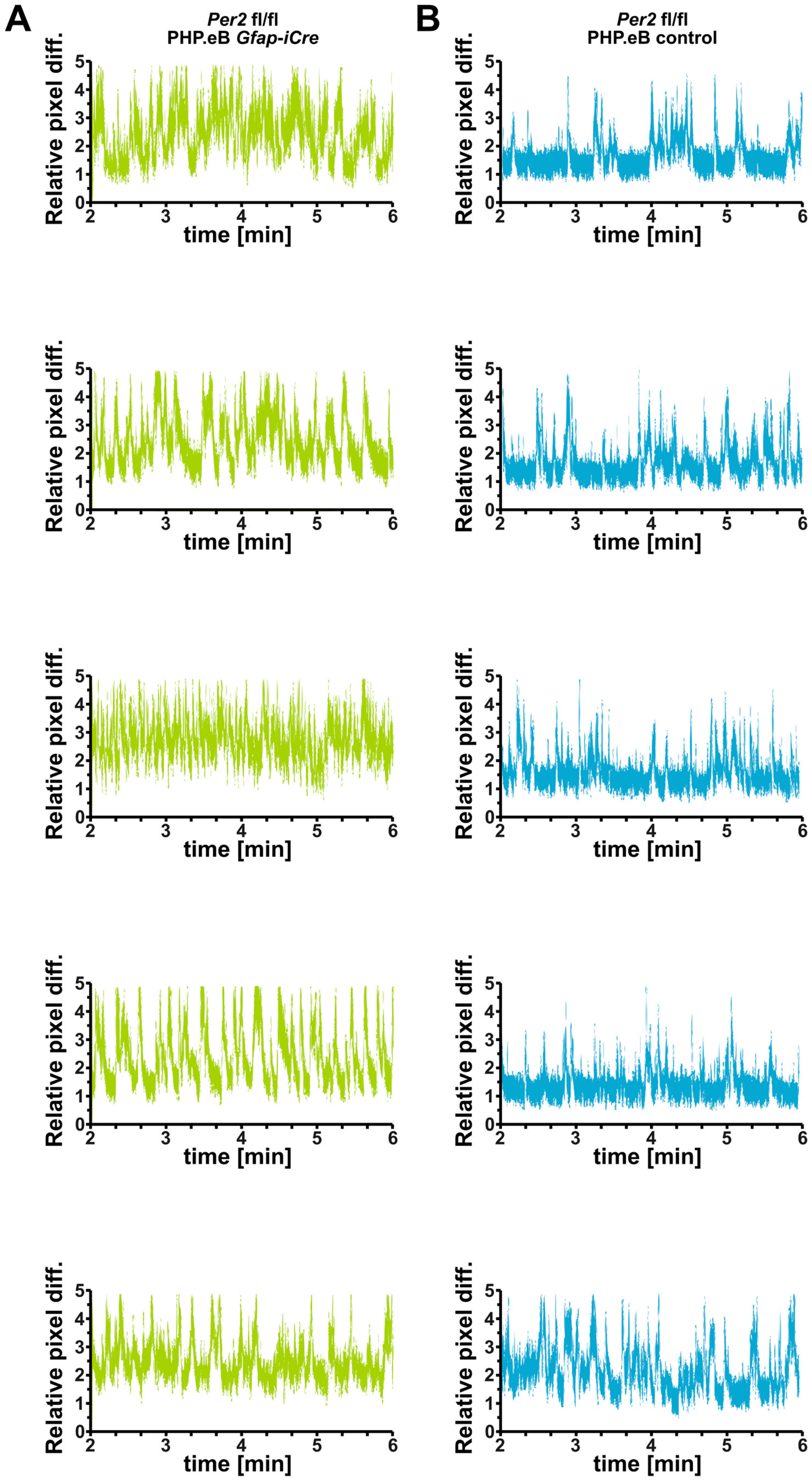
A
Per2 fl/fl
PHP.eB Gfap-iCre
B
Per2 fl/fl
PHP.eB control
Relative pixel diff.
time [min]

Suppl. Fig. 1

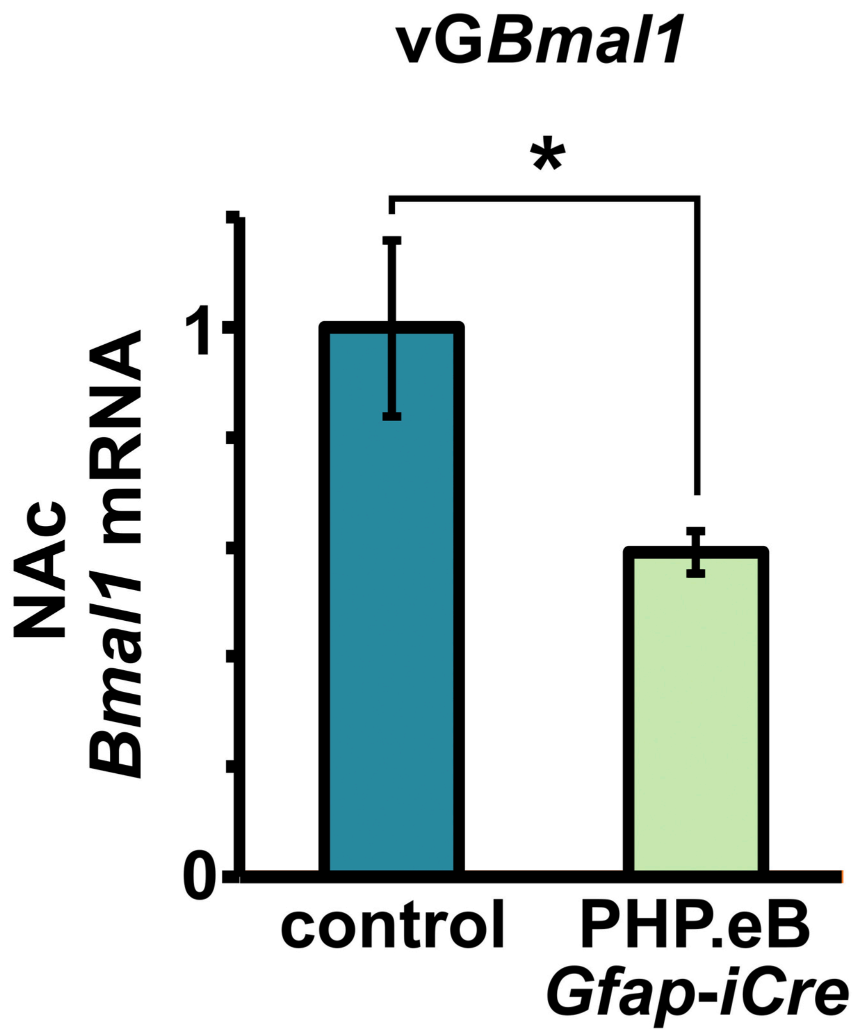

Suppl. Fig. 2

Supplemental Table 1

		Progeny										
		Cre +/-			*Cre* -/-				*Cre* +/-	*Cre* -/-		
**Mating**	***Cre* carrier**	**fl/fl**	**fl/Dfl**	**total**	**fl/fl**	**fl/Dfl**	**total**	**n**	**% Dfl**	**% Dfl**	**Difference**	**Notes**
26	female	10	27	37	18	25	43	80	72.97	58.14	14.83	
29	male	1	20	21	22	0	22	43	95.24	0.00	95.24	
33	male	1	2	3	5	0	5	8	66.67	0.00	66.67	
36	male	3	15	18	24	0	24	42	83.33	0.00	83.33	
38	male	6	4	10	19	0	19	29	40.00	0.00	40.00	
41	male	1	2	3	6	0	6	9	66.67	0.00	66.67	
44	male	7	17	24	15	0	15	39	70.83	0.00	70.83	
45	male	35	14	49	41	0	41	90	28.57	0.00	28.57	1 discarded (inconclusive genotype)

M carrier	132	260	64.47	0.00
F carrier	43	80	72.97	58.14

Supplemental Table 2

		nucleus accumbens			amygdala			medial prefrontal cortex			hypothalamus			
gene	full name	rel. Δ vs. WT *	p-value **	N per group	rel. Δ vs. WT *	p-value **	N per group	rel. Δ vs. WT *	p-value **	N per group	rel. Δ vs. WT *	p-value **	N per group	smallest p-value
Gat2/Slc6a13	GABA transporter 2	1.67200	0.00792	6	0.84251	0.65663	3	1.12610	0.42204	3	0.49669	0.15719	3	0.00792
Eaat1	excitatory amino acid transporter 1	1.04613	0.85813	6	0.95439	0.76588	3	1.02759	0.95246	3	0.99692	0.97981	3	0.76588
Eaat2	excitatory amino acid transporter 2	0.92958	0.66133	6	0.90787	0.57846	3	1.03055	0.91148	3	0.87722	0.12746	3	0.12746
Maoa	monoamine oxidase A	0.99121	0.95198	6	1.01672	0.93794	3	1.00434	0.98245	3	1.00145	0.99466	3	0.93794
Maob	monoamine oxidase B	0.94880	0.76553	6	0.91038	0.18386	3	1.08833	0.67899	3	1.12360	0.28016	3	0.18386
Gabra1	GABA receptor subunit alpha-1	0.97835	0.90355	6	0.95540	0.69682	3	0.92147	0.76567	3	0.92538	0.50802	3	0.50802
Gabra2	GABA receptor subunit alpha-2	1.14240	0.28715	6	1.51702	0.05586	3	1.52571	0.10686	6	1.45088	0.11052	3	0.05586
Gat1	GABA transporter 1	0.97738	0.94776	6	1.15281	0.58424	3	1.13179	0.51951	3	0.49651	0.02529	3	0.02529
Aadc	aromatic L-amino acid decarboxylase	1.47268	0.52475	3	0.93074	0.81351	3	0.88371	0.54718	3	0.72737	0.31008	3	0.31008
Tph2	tryptophan hydroxylase 2	1.90991	0.21618	3	0.80914	0.51223	3	0.87692	0.74469	3	0.99168	0.95876	3	0.21618
Th	tyrosine hydroxylase	1.01326	0.96115	6										0.96115
Comt	catechol-O-methyltransferase	1.01111	0.91634	6										0.91634
Gls	glutaminase	0.99761	0.99229	6										0.99229
Glns	glutamine synthetase / glutamate ammonia ligase	1.07378	0.45718	6										0.45718
Drd1	dopamine receptor D1	1.01636	0.89078	6										0.89078
Drd2	dopamine receptor D2	1.05641	0.71733	6										0.71733
Drd3	dopamine receptor D3	2.11310	0.00081	6										0.00081
Trank1	tetratricopeptide repeat and ankyrin repeat containi	1.20527	0.40539	6										0.40539
Tac1	tachykinin precursor 1	1.01403	0.89458	6										0.89458
Per2	period 2	0.66225	0.00442	6										0.00442
Gat3/Slc6a11	GABA transporter 3	1.04751	0.93750	3	1.23453	0.18240	3	1.12030	0.66637	3	1.08946	0.68440	3	0.18240

* expression in WT is 1, therefore a reduction is < 1 and an upregulation is > 1

** two-tailed student's t-test

Supplemental Table 3

		dorsal striatum						nucleus accumbens						medial prefrontal cortex					
neurotransmitter	full name	ng/mg C	ng/mg KO	SEM C	SEM KO	p-value *	N per group	ng/mg C	ng/mg KO	SEM C	SEM KO	p-value **	N per group	ng/mg C	ng/mg KO	SEM C	SEM KO	p-value **	N per group
GABA	gamma-aminobutyric acid	368.04	391.55	91.84	71.25	0.87	5 C, 3 KO	298.51	286.44	48.58	35.57	0.85	5 C, 5 KO	75.00	75.51	5.16	4.25	0.94	8 C, 7 KO
glu	glutamate	1316.92	1241.12	25.78	44.87	0.52	5 C, 3 KO	1361.26	1257.97	32.54	25.81	0.04	5 C, 5 KO	1676.71	1623.11	26.62	23.13	0.16	10 C, 8 KO
gln	glutamine	772.05	829.05	65.54	51.18	0.57	5 C, 3 KO	834.40	785.39	36.01	31.88	0.34	5 C, 5 KO	618.15	624.59	20.34	20.96	0.83	10 C, 8 KO

SEM standard error of mean
* two-tailed student's t-test

Supplemental Table 4

Aadc

FW: CAT GAG AGC TTC TGC CCT TCG G
RV: GCA GGA TGT GGT CCC CAG TGT
TM: CGG GAC AAG GCA GCT GGC CTG ATT CCA

Comt

FW: GTG GCT ACT CAG CCG TGC GA
RV: GCT GGG TGA TGG CAG CGT AGT
TM: TGG CCC GCC TGC TGC CAC CT

Ddc

FW: AGA AGA ACT GGT GTG AGG AGC AGT
RV: TGC CTG CAG CTG GCG GAT AA
TM: TGG TGG CCC TAC TGG CTG CTC GGA

Drd1

FW: AGG AGA GGG CGC AGG GTT G
RV: GCC CCT GGT GCC ACA TCT CT
TM: CGG AGT CGG GGA GCG TGG TCT CCC

Drd2

FW: GAT GCG GCG GGA GCT GGA A
RV: TGG GTG GCA CGG CTC TTC AA
TM: TCT CTG GCC CCG GGC GCC CT

Drd3

FW: CCT GGC TTC CCT CAG CAG TCT
RV: GCT CCA TTT GTC CCG TGG CAT CT
TM: TGT CTG CGG CTG CAT CCC ATT CGG CA

Eaat1

FW: GAT GCT GGT CTT GCC CCT GAT
RV: ACA GCG CGC ATC CCC ATC TT
TM: CCA GTC TCG TCA CAG GAA TGG CGG CCC

Eaat2

FW: ACA GGG TTG TCA GGC CTG GAT
RV: CAG CAC GGC GGC AAT GAT GG
TM: AGC CAG CGG CCG CCT AGG CA

Gabra1

FW: GTC TGG AGC GAT CCG GTG C
RV: TGA GGG TCC AGG CCC AAA GA
TM: CCC GAG CTG TGC AAG CCC GTG ATG A

Gabra2

FW: TGC AAT GTA TGG TCT CTG CTG CTT GT
RV: AGC CTC ATC TTC TTG GAT GTT AGC CA
TM: TGG TGT GGG ACC CAG TCA GGT TGG TGC

Gat1

FW: CGT GGA ACA CTG ACC GCT GCT
RV: GGT GCA TGT TGC GCT CCC AGA
TM: CCA CCA ACA TGA CCA GCG CCG TGG TGG

Gat2/Slc6a13

FW: CTG GGA GAG GCG AGT CCT GA
RV: GCA GGA GGC ACA GGA CCA GTT
TM: CGG ATG GCA TCC AGC ACC TGG GGT CC

Glns

FW: TGG ACC CCA AGG CCC GTA T
RV: ACA AGC AGG CCC GGT AGT GA
TM: AGG CCT TGT CTG CTC CCA CAC CGC A

Gls

FW: TGT CTG CCC TCC GAA GGT TTG C
RV: ACC CTC TGC TGC TGC GAC AT
TM: CTG TCA GCC ATG GAC ATG GAG CAG CGG G

iCre

FW: GGG TTA CCA AGC TGG TGG AG
RV: GGC AGC CAC ACC ATT CTT TC

MaoA

FW: GGT ATG TGA GGC AGT GTG GAG GT
RV: CAC TTA TTT GGC CAG AGC CAC CT
TM: CAG TCA CCA ATG GCG GCC AGG AAC GGA

MaoB

FW: TGG AGC GGC TAC ATG GAG GG
RV: TCT GGA ATC TTC CCA ATG GCA TGA AGA

TM: TGG AGG CTG GGG AGA GAG CAG CCA

Per2

FW: TCC ACA GCT ACA CCA CCC CTT A
RV: TTT CTC CTC CAT GCA CTC CTG A
TM: CCG CTG CAC ACA CTC CAG GGC G

Tac1

FW: AGA GCA AAG AGC GCC CAG CA
RV: CGC CAC GGC CAC GAG GAT TT
TM: CCT GCG GAG CAT CCC CGC GG

Th

FW: CCT GGA CCA TCC GGG CTT CT
RV: GGG GAA TTG GCT CAC CCT GCT T
TM: CCA GGC GTA TCG CCA GCG CCG G

Tph1

FW: ACT GCG ACA TCA GCC GAG AAC A
RV: TTC GCA GTG AGC TGA TCG GG
TM: ACG CCA CCG TCC TCT CGG TGG ACT C

Tph2

FW: ACC CAG TAC GTG CGG CAT GG
RV: GCA GTG GCA CGT GTC CCA AGA
TM: CCG ACC CCC TCT ACA CCC CGG AAC C

Trank1

FW: GGT GTC ACC TGC GCG CAT CC
RV: GCC AGT TCC CGA GGA GGA GT
TM: CGG CGG CGA GTC CCG GCC AT

Tspo

FW: GGT CAG CTG GCT CTG AAC TG
RV: CAG TCG CCA CCC CAC TGA CA
TM: TGC CCG GCA GAT GGG CTG GGC
